# Hybridization Capture-Based Next-Generation Sequencing to Evaluate Coding Sequence and Deep Intronic Mutations in the *NF1* Gene

**DOI:** 10.3390/genes7120133

**Published:** 2016-12-17

**Authors:** Karin Soares Cunha, Nathalia Silva Oliveira, Anna Karoline Fausto, Carolina Cruz de Souza, Audrey Gros, Thomas Bandres, Yamina Idrissi, Jean-Philippe Merlio, Rodrigo Soares de Moura Neto, Rosane Silva, Mauro Geller, David Cappellen

**Affiliations:** 1 Graduate Program in Pathology, School of Medicine, Universidade Federal Fluminense, Niterói 24033-900, Brazil; 2 Department of Pathology, School of Medicine, Universidade Federal Fluminense, Niterói 24033-900, Brazil; 3 Neurofibromatosis National Center (Centro Nacional de Neurofibromatose), Rio de Janeiro 20011-330, Brazil; 4 Anatomy Pathology Service, Hospital Universitário Antônio Pedro, Universidade Federal Fluminense, Niterói 24033-900, Brazil; nsoliveira95@gmail.com (N.S.O.); anna.karol@gmail.com (A.K.F.); 5 School of Biomedicine, Universidade Federal Fluminense, Niterói 24210-130, Brazil; ccruz@id.uff.br; 6 Service de Biologie des Tumeurs, Centre Hospitalier Universitaire de Bordeaux, Hôpital du Haut Lévêque, Pessac F-33604, France; audrey.gros@chu-bordeaux.fr (A.G.); thomas.bandres@chu-bordeaux.fr (T.B.); jp.merlio@u-bordeaux.fr (J.-P.M.); david.cappellen@u-bordeaux.fr (D.C.); 7 Inserm (Institut National de la Santé et de la Recherche Médicale) U1053, Bordeaux Research in Translational Oncology (BaRITON) and University of Bordeaux, Bordeaux F-33076, France; yamina.idrissi@u-bordeaux.fr; 8 Biology Institute, Universidade Federal do Rio de Janeiro, Rio de Janeiro 21941-599, Brazil; rsmouraneto@gmail.com; 9 Carlos Chagas Filho Biophysics Institute, Universidade Federal do Rio de Janeiro, Rio de Janeiro 21941-599, Brazil; silvaros@biof.ufrj.br; 10 Department of Immunology and Microbiology, School of Medicine, Centro Universitário Serra dos Órgãos, Teresópolis 25964-004, Brazil; maurogeller@gmail.com; 11 Martagão Gesteira Child Care and Pediatrics Institute, Universidade Federal do Rio de Janeiro, Rio de Janeiro 21941-912, Brazil

**Keywords:** Neurofibromatosis 1, *NF1* gene, next generation sequencing

## Abstract

Neurofibromatosis 1 (NF1) is one of the most common genetic disorders and is caused by mutations in the *NF1* gene. *NF1* gene mutational analysis presents a considerable challenge because of its large size, existence of highly homologous pseudogenes located throughout the human genome, absence of mutational hotspots, and diversity of mutations types, including deep intronic splicing mutations. We aimed to evaluate the use of hybridization capture-based next-generation sequencing to screen coding and noncoding *NF1* regions. Hybridization capture-based next-generation sequencing, with genomic DNA as starting material, was used to sequence the whole *NF1* gene (exons and introns) from 11 unrelated individuals and 1 relative, who all had NF1. All of them met the NF1 clinical diagnostic criteria. We showed a mutation detection rate of 91% (10 out of 11). We identified eight recurrent and two novel mutations, which were all confirmed by Sanger methodology. In the Sanger sequencing confirmation, we also included another three relatives with NF1. Splicing alterations accounted for 50% of the mutations. One of them was caused by a deep intronic mutation (c.1260 + 1604A > G). Frameshift truncation and missense mutations corresponded to 30% and 20% of the pathogenic variants, respectively. In conclusion, we show the use of a simple and fast approach to screen, at once, the entire *NF1* gene (exons and introns) for different types of pathogenic variations, including the deep intronic splicing mutations.

## 1. Introduction

Neurofibromatosis 1 (NF1; MIM: 162200) is an autosomal dominant disorder with a complete penetrance and highly variable expressivity. It is one of the most common genetic disorders with an incidence of 1 case in 2000 births [1]. Neurofibromas, Lisch nodules, café-au-lait spots, as well as inguinal and axillary “freckles” develop in the majority of individuals with NF1. Other important features include learning disabilities, optic gliomas, specific bone lesions, and risk for the development of malignant neoplasms, mainly the malignant peripheral nerve sheath tumors (MPNSTs) [2].

NF1 is caused by a spectrum of mutations that affect the *NF1* gene, which is a tumor suppressor gene located at chromosome 17q11.2. It spans 282 kb and comprises 60 exons (57 constitutive and 3 alternatively spliced exons), being one of the largest human genes [3]. The *NF1* gene presents one of the highest frequencies of spontaneous mutations in the human genome. Therefore, half of individuals with NF1 present no family history of the disorder [4].

To date, according to the online Human Gene Mutation Database (data accessed on November 2016), more than 2600 different *NF1* mutations dispersed throughout the gene have been reported and include missense/nonsense, splicing, small insertions or deletions (indels), and also gross chromosomal alterations. Although no true hotspots have been identified in the *NF1* gene, recurrent mutations are not uncommon in individuals with NF1 [5].

Identification and characterization of *NF1* gene mutations represent a considerable research and diagnostic challenge because of its large size, existence of a number of highly homologous pseudogenes located throughout the human genome (in chromosomes 2, 12, 14, 15, 18, 21, and 22), absence of mutational hotspots as well as the diversity of mutation types, including deep intronic splicing mutations [6,7]. The *NF1* gene mutation detection rate in NF1 patients who meet the clinical diagnostic criteria is reported to be 50%–95% (reviewed by Upadhyaya [8]). The highest mutation detection rate reported so far (≈95%) is based on a multistep mutation analysis at the genomic and RNA level [9]. It includes a massive PCR genomic DNA (gDNA)/complementary DNA (cDNA) preparation and Sanger sequencing of multiple regions of the *NF1* gene, which are associated with other methodologies to identify copy number alterations (high-performance liquid chromatography, multiplex ligand probe amplification (MLPA), microsatellite marker analysis, and fluorescence in situ hybridization (FISH)).

Therefore, current NF1 molecular diagnostic testing based on Sanger sequencing is labor-intensive, time-consuming (usually takes 3–4 weeks on average), and expensive [3,8]. Moreover, even using an exhaustive analysis, mutation screening tests do not achieve a sensitivity of 100%. Possibly many, if not all, cases of patients with classical NF1, with no *NF1* mutation detected in the genetic tests, are related to mutations located in *NF1* gene regions that are not commonly screened (e.g., the flanking 5′- and 3′-untranslated regions (UTRs)) [8]. It is also possible that the disease-causing pathogenic variations are related to deep intronic mutations [8], which are missed when traditional gDNA-based screening techniques, that sequence only the exonic regions and conserved splice sites, are applied.

In the last few years, DNA sequencing has been passing through a revolution with the use of next-generation sequencing (NGS). It permits a fast and simple sequencing, making possible the analysis of large genomes. With NGS, rather than just sequence the coding exons and conserved splice sites, it is possible to screen the entire sequence of a target large gene from a gDNA sample, including the intronic regions. Therefore, NGS seems to be a very useful tool to sequence the *NF1* gene [10]. In this study, we aimed to evaluate the use of hybridization capture-based next-generation sequencing to screen not only coding, but also noncoding *NF1* regions.

## 2. Materials and Methods

This study was approved by the ethical committee of Antônio Pedro University Hospital of Universidade Federal Fluminense, Niterói, Brazil (#121/11).

### 2.1. Patients

Some clinical features of the participants of this study are shown in Table 1. NGS samples were obtained from 12 individuals with NF1: 11 unrelated NF1 patients and one relative with NF1 (NF.85; son of the patient NF.84). For the Sanger methodology (see Section 2.6), we have also included another three relatives with NF1, as follows: NF.14 (cousin of patient NF.84), NF.87 (son of patient NF.91), and NF.27 (sister of patient NF.26). All the individuals had NF1 according to the National Institutes of Health (NIH) clinical criteria [11].

Extraction of DNA from leukocytes and purification were performed with QIAamp DNA Blood Mini kit (Qiagen, Valencia, CA, USA), according to the manufacturer’s instructions. Purity of DNA was assessed by spectrophotometry using NanoDrop 2000 (Thermo Fisher Scientific, Waltham, MA, USA). DNA yields were determined using a double stranded DNA (dsDNA) broad range (BR) kit on a Qubit^®^ fluorometer (Thermo Fisher Scientific). Integrity of DNA was evaluated by agarose-gel electrophoresis.

### 2.2. Capture Probe Set Design

Capture probe set was designed and synthesized by Thermo Fisher Scientific for the Ion TargetSeq Custom Enrichment kit (Thermo Fisher Scientific) to target all the coding exons, UTRs, as well as the intronic regions of the *NF1* gene. The designed nonoverlapping probe set covered 90.5% of the targeted region (chr17: 29421944-29704695).

### 2.3. Targeted Capture Library Construction and Next-Generation Sequencing

Libraries were constructed from 2–3 µg of DNA. DNA was sheared into 200 bp fragments on a Bioruptor^®^ sonicator (Diagenode, Denville, NJ, USA), using two cycles of 15 min. Then, DNA fragments were end-repaired, nick repaired, and the barcodes and adaptors were ligated to fragmented DNA. After, we size-selected and amplified the library using the IonXpress Plus gDNA Fragment Library kit, Ion Xpress Barcode Adapters and E-Gel Size Select 2% agarose gels (Thermo Fisher Scientific), according to the manufacturer’s instructions. The amplified library was then hybridized to the *NF1* capture probe set for two days (two hybridizations). Libraries were quantified in Qubit^®^ fluorometer (Thermo Fisher Scientific) with dsDNA high sensitivity (HS) kit. The quantity and quality of the library were also verified with Agilent Bioanalyzer DNA High Sensitivity kit (Agilent, Richardson, TX, USA). Libraries were then pooled in equimolar concentrations and diluted to 26 pM, prior to clonal amplification onto Ion Sphere Particles using the Ion One Touch 2 System and the Ion OneTouch 200 Template kit v2 (Thermo Fisher Scientific), following the manufacturer’s instructions. Coated spheres were enriched on the Ion Torrent ES (Thermo Fisher Scientific) before loading onto Ion 316 or 318 sequencing chips (Thermo Fisher Scientific). NGS was performed on Ion Torrent Personal Genome Machine—Ion Torrent PGM™ (Thermo Fisher Scientific).

### 2.4. Bioinformatic Analysis of Next-Generation Sequencing Data

Primary analyses of NGS data were performed using Torrent Suite software on the Ion Torrent Browser (Thermo Fisher Scientific): base calling, quality score assignment, adapter trimming, and demultiplexing. Reads were generated using Ion Torrent platform pipeline software, which includes standard control of the read quality. Using the same software, reads were aligned against human whole genome (hg19). Only high-quality reads (Q ≥ 20) were aligned to the reference sequence using TMAP (Torrent Mapping Alignment Program).

Germline variants in the targeted regions were detected using the variant caller plug-in of the Ion Torrent Suite software. Variant caller was run with default germline low stringency settings. For each sample, all variants were exported through a vcf (Variant Call Format) file. Variants were filtered following the criteria of a minimum coverage of 30×, and a sample variant frequency between 30%–70%. For variant interpretation, Ingenuity^®^ Variant Analysis™ software (Qiagen) and SNPnexus web server were used. Variants were also viewed manually on Integrative Genomics Viewer (IGV—version 2.3.40; Broad Institute, Cambridge, MA, USA) and on Alamut^®^ Visual (version 2.6.1e; Interactive Biosoftware, Rouen, France) to eliminate strand bias and reduce false positive calls.

### 2.5. In Silico Predictions

To predict the potential effect of the mutation on the structure and function of the protein codified by the *NF1* gene (neurofibromin), PolyPhen2 and SIFT were applied through Ingenuity^®^ Variant Analysis™ software and SNPnexus web server. In silico splicing predictions were investigated with Alamut^®^ Visual, which uses the following prediction methods: SpliceSiteFinder-like; MaxEntScan; NNSPLICE; GeneSplicer; Human Splicing Finder; Known Constitutive Signals; ESEFinder; RESCUE-ESE. Because Alamut^®^ Visual integrates only Human Splicing Finder’s prediction capabilities on donor and acceptor splice sites, and does not investigate the mechanisms of the splicing abnormalities caused by mutations at the nonconsensus splice sites, we also used the online Human Splicing Finder (http://www.umd.be/HSF/).

### 2.6. Confirmatory Sanger Sequencing

All variants were confirmed by Sanger sequencing in an AB3130 Genetic Analyzer (Applied Biosystems, Carlsbad, CA, USA). Primers were designed using NCBI’s Primer BLAST tool (Table 2) and the sequences were analyzed with the Sequencing Analysis software (version 5.4; Applied Biosystems).

## 3. Results

### 3.1. Sequencing Statistics

The average number of bases with Q ≥ 20 across the three Ion Torrent PGM^TM^ runs (12 samples) was 96.9 Mbp, constituting 83.3% of total output. Overall average depth was 177, ranging from 52 to 470. The alignment parameters of the reads for each sample and for each run are shown in Table S1. Average coverage depth for all *NF1* exons and introns is shown in Table S2. With the exception of exon 1, which had an average coverage depth of 9.5×, all the other *NF1* exons were highly covered, with an average coverage depth ranging from 68.5× to 280.4×. The average coverage depth of the intronic regions ranged from 46.1× to 228.4×. The distribution of reads into different chromosomes is shown in Table 3. About 84% of the reads were mapped to chromosome 17, which contains the *NF1* gene.

### 3.2. Mutation Detection

**Figure 1:**
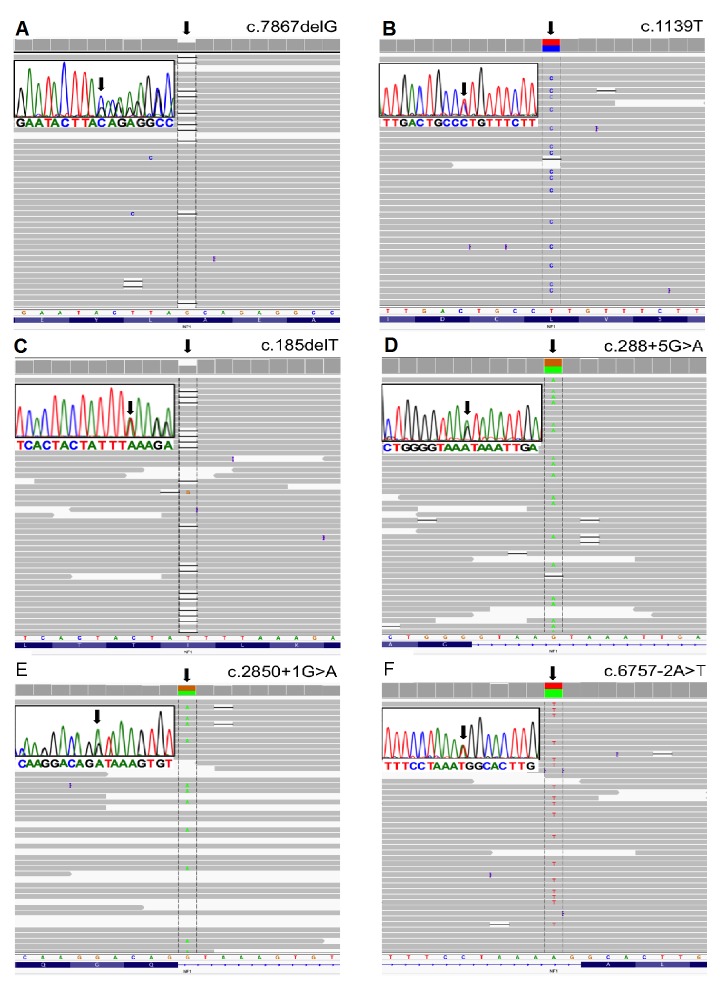
Mutations found using next-generation sequencing (NGS) and illustrated in Integrative Genomics Viewer (IGV). Inserts: Sanger sequencing of the corresponding mutations: (**A**) Patient 1—c.7867delG; (**B**) Patient 2—c.1139T > C; (**C**) Patient 3—c.185delT; (**D**) Patient 4—c.288 + 5G > A; (**E**) Patient 5—c.2850 + 1G > A; (**F**) Patient 6—c.6757 − 2A > T.

**Figure 2:**
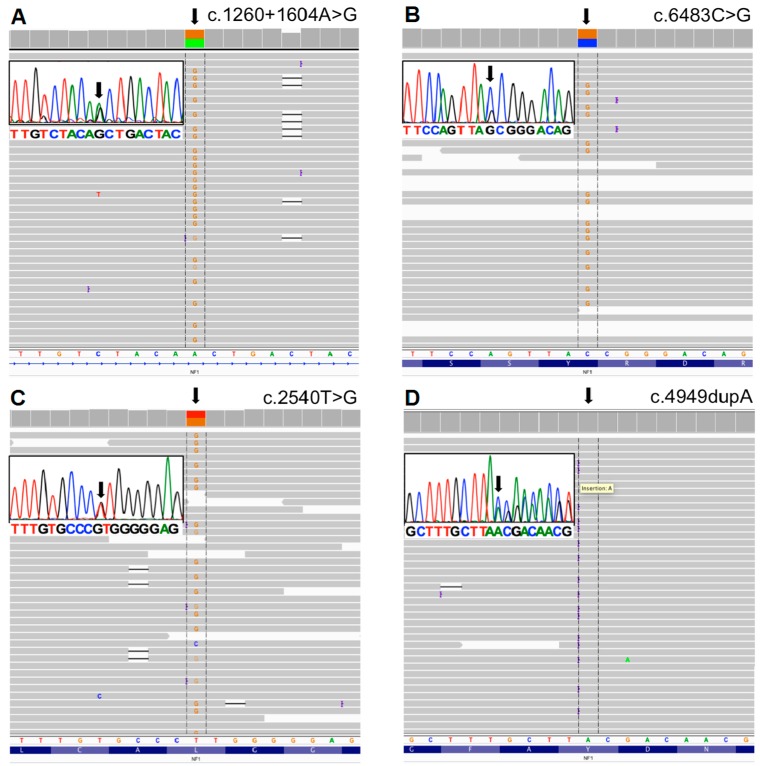
Mutations found using NGS and illustrated in IGV. Inserts: Sanger sequencing of the corresponding mutations: (**A**) Patient 7—c.1260 + 1604A > G; (**B**) Patient 8—c.6483C > G; (**C**) Patient 9—c.2540T > G; (**D**) Patient 10—c.4949dupA.

To detect the pathogenic variants, we blindly inspected all mapped sequence reads from NGS from the 12 samples (11 unrelated patients). Following variant filtering, a total of 3554 variants were identified (≈238/patient; Table S1). With the criteria of minimum coverage of 30×, annotation and filtering of the variants resulted in correct interpretation of the pathogenic variation, as confirmed by Sanger sequencing (Figure 1 and Figure 2), except for one case (NF.19) where no mutation was identified with NGS. In this case, since exon 1 had low coverage, it was sequenced by Sanger method, but no mutation was found (primers are shown in Table 2).

Considering only the 11 unrelated NF1 patients, our approach showed a mutation detection rate of 91% (10 out of 11). We identified eight recurrent and two novel mutations. A summary of the mutations found in this study is shown in Table 4. As expected, the same mutations were found with NGS in the samples from patients NF.84 and NF.85, who were from the same family. Sanger sequencing showed the same mutations in patients NF.14 and NF.84, NF.87 and NF.91, as well as NF.27 and NF.26, who were also from the same family.

Point mutations accounted for 100% of the 10 *NF1* pathogenic variants identified in this study. Splicing alterations (c.288 + 5G > A; c.1260 + 1604A > G; c.6483C > G; c.2850 + 1G > A; c.6757 − 2A > T) accounted for 50% (5 out of 10) of the mutations. All were recurrent, with the exception of the mutation c.288 + 5G > A; the splicing effect was predicted by Alamut^®^ Visual. For the mutation c.6483C > G, Alamut^®^ Visual predicted that the reading frame was interrupted by a premature stop codon. As demonstrated by Ars et al. [12], at the mRNA level, this is a nonsense mutation with a splicing effect, which leads to an exon skipping. Therefore, it was classified as a splicing mutation. One of the identified splicing alterations (c.1260 + 1604A > G) was caused by a deep intronic mutation located in intron 11 (1604 bp from exon 10).

Truncating mutations (c.185delT c.4949dupA; c.7867delG) corresponded to 30% (3 out of 10) of the pathogenic variants. The mutation c.185delT was already described by Forzan et al. [13]. This is a mutation that interrupts the reading frame prematurely; codon L62 is replaced by a STOP codon (p.L62*). The mutation c.4949dupA was previously described by Mattocks et al. [14] and van Minkelen et al. [15]. This duplication interrupts the reading frame prematurely: codon Y1650 is replaced by a STOP codon (p.Y1650*). The mutation c.7876delG is novel and, as predicted by Alamut^®^ Visual, creates a frame shift starting at codon A2623. The new reading frame ends in a STOP codon 34 nucleotides downstream (p.A2623Qfs*35). Missense mutations (c.1139T > C, causing a p.L380P leucine to proline amino acid substitution; c.2540T > G, leading to a p.L847R leucine to arginine amino acid substitution) accounted for 20% (2 out of 10) of the pathogenic variants and were all recurrent.

## 4. Discussion

We developed an approach to analyze the entire *NF1* gene, including the exonic and intronic regions by hybridization capture-based NGS. Although some previous studies had evaluated the use of NGS technology to investigate mutations in the *NF1* gene [3,7,16,17,18,19,20,21], only exons and conserved splice sites were sequenced, which is the approach commonly used by other investigators based on Sanger methodology.

In our study, the choice of using a hybridization-based targeted selection to prepare the gDNA library, instead of using PCR-based enrichment to extract the *NF1* region for sequencing, was based on its ability to capture a large target region in a single experiment, more conveniently, with less hands-on time [22]. We could design a probe set that covered 90.5% of the target bases of the *NF1* gene. Most (99.3%) of the bases of the *NF1* gene not covered by the designed probe set were located outside the exonic regions. Therefore, there was a small percentage of the exonic regions that was not covered by the capture probe set.

The average of the percentage of the target covered at least once and ≥30× was 97.7% and 96.0%, respectively, showing an excellent capture performance. Also, we were able to reach reads in the 150 bp range with total sequence throughput that guaranteed high coverage depth, which is important to achieve high sensitivity, preventing false-positive variant calls [23]. We identified 10 different pathogenic variations in 11 unrelated patients with clinical diagnosis of NF1, which represents a mutation detection rate of 91%. This high mutation rate is similar to the results of other studies that had used an exhaustive multistep methodology [9,24].

All exons and introns of the *NF1* gene had a high depth of coverage with exception of exon 1. As it occurs in other genes, the first exon of the *NF1* gene presents higher GC content (71%), compared to the other exons (42%) [7]. It is known that coverage may be reduced in regions with GC contents outside of an optimum range of 40%–60% [25,26], and low coverage in exon 1 of the *NF1* gene had been previously reported in other studies with NGS, using both PCR-based enrichment and hybridization-based targeted selection of the *NF1* gene for library preparation [7,19]. Regarding the use of PCR-based enrichment, high/low GC content reduces the efficiency of PCR amplification. With respect to the hybridization-based targeted selection, the problem is a reduced efficiency of capture probe hybridization to sequences with high/low GC content [26]. The latter is a property of the capture probes, and therefore with the advance of technologies, it is expected that, to some extent, this limitation is going to be compensated by a better probe set design [26], which would substantially improve the performance of our approach. Sequencing the exon 1 of the *NF1* gene is also a challenge in cDNA-based protocols with Sanger methodology [24].

In the only case in which no mutation was found using NGS, we sequenced the coding region of exon 1 by Sanger method. Nevertheless, no pathogenic variation was found. It is possible that the disease-causing mutation was located outside exon 1, in regions of the *NF1* gene not covered by the designed probe set or in regions with <30× of coverage depth. As suggested in the literature, in cases of patients with NF1 with no *NF1* gene mutation detected, it is possible that the disease is caused by increased methylation of the *NF1* promoter region, which would downregulate gene expression [6]. There is also the possibility that the mutation was a single or multiexon deletion or even a total deletion (microdeletion) of the *NF1* gene, which are not easily detectable by our approach. Literature reports that ≈5% of *NF1* mutations are microdeletions, which have been detected by FISH, MLPA analysis, and/or array comparative genomic hybridization (array-CGH) [2]. Moreover, ≈2.5% of all mutations found in the *NF1* gene are small copy-number changes involving one to multiple exons detectable by long-range reverse transcription polymerase chain reaction (RT-PCR) and/or MLPA [2]. Although NGS has the potential to detect copy-number variations, its use for this aim has been limited by a lack of available and effective statistical approaches [29,30]. We believe that in the future, with improvement of bioinformatics tools, NGS will eliminate the necessity for using other methodologies to evaluate these types of mutations in the *NF1* gene.

Another challenge of sequencing the *NF1* gene is the high number of nonfunctional pseudogenes throughout the human genome. Because pseudogenes are not subject to selective pressure, their mutation rate is much higher than that of the functional genes from which they originate, and therefore their unintended sequencing can lead to false positive variant calls [31]. Target enrichment strategies and data analysis are particularly important to avoid interference of pseudogenes in NGS, allowing reliable detection of true pathogenic variations [31]. In our study, in order to avoid forcing alignment of possible amplified pseudogene regions on the *NF1* sequence, reads were aligned against the whole human genome and only high-quality reads (Q ≥ 20) were aligned to the reference sequence. Sanger sequencing confirmed all the mutations identified by NGS, showing that there were no false positive variants from pseudogenes reads.

Naturally, with our approach, the number of total variants that were identified was high (average of 238 variants/patient) compared to other studies that have focused on the exonic regions (average of 30 variants/patient) [3]. Therefore, the use of in silico prediction tools was important to filter out the variants unlikely to be harmful, restricting the search to fewer candidate variants for more detailed manual inspection. Due to the filtering strategy we used, the size of the sequenced region was not an obstacle since only few variants were selected as final candidates.

Homopolymer errors are the dominant error type in Ion Torrent PGM^TM^ data. By manually checking all possible pathogenic variants calls with the IGV software, we were able to identify some as homopolymer artifacts and thus they did not lead to false positive results.

Most (80%) of the mutations identified in this study were recurrent and two (20%) were new. Splicing defects accounted for 40% of the mutations in our sample series. In the literature, approximately 29% of all reported mutations in the *NF1* gene cause aberrant splicing and over 57% of them reside outside the canonically conserved GT-AG dinucleotides at the splice donor and acceptor sites [32]. Moreover, around 10% of all splicing defect-causing mutations in the *NF1* gene belong to the class of deep intronic mutations (located >500 bp away from exon boundaries), representing 1%–3% of all *NF1* mutations [27,32,33,34]. The effect of deep intronic mutations on mRNA is the inclusion of a part of an intron due to the creation of a novel donor or acceptor site or an exonic splicing enhancer site, which, together with a nearby cryptic splice, defines a cryptic exon that is incorporated into the mature mRNA by the spliceosome [35].

Based on the high prevalence of splicing mutations in the *NF1* gene, some authors propose the use of mRNA/cDNA for mutation detection, which also identifies deep intronic splicing mutations [32]. Over the past decades, virtually all reported deep intronic mutations in different target genes had been identified through mRNA analysis [10]. Nevertheless, mRNA-based assays present challenges, and precautions need to be taken to prevent problems inherent in this approach [6,10,24]. For example, short-term phytohemagglutinin-stimulated lymphocyte cultures are used to avoid illegitimate splicing that leads to multiple aberrant splice variants that impede the detection of mutations in an RNA-based approach. Moreover, before mRNA extraction, cultures should be treated with puromycin to prevent nonsense-mediated mRNA decay.

The use of gDNA is much easier in clinical settings in comparison to cDNA. Nevertheless, the approach that has been used in the literature to analyze the *NF1* gene based on gDNA only sequences the exons and conserved splice sites, which span ≈5 kb, and therefore does not identify deep intronic mutations. Of course, it would be impracticable in clinical settings to use Sanger methodology to sequence the whole *NF1* gene, including intronic and exonic regions, which spans ≈282 kb. One great advantage of the NGS technology associated with the in silico prediction tools is the capacity to analyze the entire intronic sequence of a target gene, making possible the identification of deep intronic mutations from a gDNA sample. Nowadays, there are a number of in silico tools that have been developed to assess the effect of DNA sequence variations on splicing, including deep intronic mutations. The online Human Splicing Finder correctly predicted the activation of an exonic cryptic acceptor site in one of our samples (c.1260 + 1604A > G). This deep intronic mutation is recurrent and was reported in three previous studies [15,24,27]. Its consequence had been previously demonstrated by Sabbagh et al. [27] who identified, by a combined cDNA/gDNA sequencing strategy, that this mutation causes a creation of an acceptor splice site, leading to an insertion of a cryptic exon (42 bp out of frame).

Currently, on a routine basis, there is a need to confirm the in silico-predicted splicing alterations [36]. Follow-up analysis of cDNA would be the simplest tool for this. Nevertheless, other techniques can also be used for this purpose using gDNA (i.e., in vitro splicing assay and minigene splicing assay [13,37]). We did not confirm the effect of any of the splicing mutations found in our sample. The majority of them (4/5) were recurrent and the splicing alteration effect had already been previously confirmed by other studies. One of the splicing mutations in our series is novel (c.288 + 5G > A). Mutation at this position with a possible splicing alteration effect (c.288 + 5G > C) had been previously reported [15]. The other novel pathogenic variant identified in our study (c.7867delG), as predicted by Alamut^®^ Visual, was classified as a frameshift mutation with a truncating effect.

Developing a 100% sensitive genetic test for NF1 is necessary to help to establish the diagnosis of patients who do not fulfill the NIH clinical diagnostic criteria [11], which is often the case among young patients with sporadic disease and patients with atypical manifestations or unusual combinations of features [6,16]. Moreover, identification of the pathogenic *NF1* mutation provides a presymptomatic/prenatal test in the offspring of sporadic patients [24]. In the research field, NGS will be extremely important to accelerate the rate of identification of mutations in the *NF1* gene, therefore facilitating the study of the effect of specific mutations on structure and function of the gene product, and leading to a better understanding of the correlation between genotype and phenotype.

Limitations of this study include the small sample size and also the existence of small regions of the *NF1* gene not covered by the designed probe set. Nevertheless, our results, together with the results of others that used NGS to sequence the *NF1* gene [3,16,17,18,19,20], show that this technology is applicable to *NF1* mutation screening. In this study, we show the results of a simple and fast approach, using hybridization capture-based NGS with gDNA as starting material, to screen, at once, the entire *NF1* gene (exons and introns) for different types of pathogenic variations, including the deep intronic splicing mutations. More studies with young patients with sporadic disease and patients with atypical features would be important to demonstrate the clinical utility of our approach.

## Figures and Tables

**Table 1:** Summary of some clinical features of the participants.

Patient Number	Gender	Age	Family History	Number of Skin Neurofibromas	Superficial Plexiform Neurofibroma	Number of Café-au-Lait Macules	Inguinal/Axillary Freckling
NF.01	Female	46	No	200–499	Yes	3	Yes
NF.14	Female	54	Yes	500–1000	Yes	20	Yes
NF.19	Female	26	No	500–1000	No	35	Yes
NF.26	Male	52	Yes	>1000	No	3	Yes
NF.27	Female	55	Yes	>1000	No	4	Yes
NF.42	Male	47	No	200–499	No	9	Yes
NF.54	Female	32	No	200–499	No	12	Yes
NF.63	Female	48	Yes	500–1000	No	12	Yes
NF.78	Female	13	Yes	<10	No	44	Yes
NF.83	Male	48	No	>1000	Yes	8	Yes
NF.84	Female	45	Yes	200–499	Yes	28	Yes
NF.85	Male	24	Yes	200–499	Yes	48	Yes
NF.87	Male	34	Yes	200–499	No	6	Yes
NF.91	Male	72	No	>1000	No	2	Yes
NF.96	Male	41	Yes	100–199	No	17	No

**Table 2:** Primers used for PCR amplification and sequencing of the neurofibromatosis (*NF1*) gene by the Sanger method.

Patient Number	Location	Sequences (5′–3′)	T_m_	Amplicon
NF.01	Intron 11	F: TAATGAGCCAGGGCATTGTACC	66 °C	385 pb
		R: CTTTCACCAAGTACACTGAGGC	66 °C	
NF.19 *	Exon 1	F: CACAGACCCTCTCCTTGCCTCTTC	71 °C	243 pb
		R: TACCTCCCCTCACCTACTCTGTCC	68 °C	
NF.26 NF.27	Exon 43	F: TGCTGTTTGGCATTAGCAAAGT	62 °C	302 pb
		R: TGTTACCAATAACACAGTCCATGC	68 °C	
NF.42	Exon 37	F: ATACCGGGCCTAGCAATCGC	64 °C	133 pb
		R: TTGGTGTACTCCCTGACCCAGG	70 °C	
NF.54	Exon 2	F: AGCAGAACACACATACCAAAGTCAG	72 °C	158 pb
		R: AATTCCCCAAAACACAGTAACCCAA	72 °C	
NF.63	Exon 3/Intron 3	F: GATGTGTGTTGATTGGTAGCAGA	66 °C	245 pb
		R: GGACTGTCCTCTTGGTCCACA	66 °C	
NF.78	Exon 54	F: CTTGGCAGGCTACACTGGT	60 °C	158 pb
		R: ACTTAAAGACAGGCACGAAGGT	64 °C	
NF.83	Exon 21	F: AAGAAATTTGACACTCGGCTGAT	64 °C	483 pb
		R: TGCTGACAGGTGTATCTGCG	62 °C	
NF.85 NF.84 NF.14	Exon 10	F: AGCTGGATTTTACTGCCATTTGTG	68 °C	233 pb
		R: TAAAGTGTTGGTTGTTGTGAGGG	66 °C	
NF.91 NF.87	Exon 21/Intron 21	F: CCTGCTCTGTATCCAATGCTAT	64 °C	133 pb
		R: GCTTATTTCAAACAAGTCACTCT	62 °C	
NF.96	Intron 45/Exon 46	F: AGCTAGCTACCAAGATCACCA	62 °C	297 pb
		R: ACACTGATACCCAAAATGAATGC	64 °C	

F: forward; R: reverse; T_m_: primer’s melting temperature (2 × (AT) + 4 × (GC)); bp: size of the amplicons in base pairs. * In this case, no pathogenic variant was identified using next-generation sequencing (NGS). Since exon 1 showed low coverage, it was sequenced using Sanger method, but no mutation was found.

**Table 3:** Percentage of reads mapped to chromosome 17, which contains the *NF1* gene, and to chromosomes that contain NF1 pseudogenes.

Chromosome	Percentage (%)
Chr2	1.72
Chr12	0.84
Chr14	1.27
Chr15	2.57
Chr17	83.94
Chr18	1.09
Chr21	1.06
Chr22	1.81
Other chromosomes	5.71

**Table 4:** Positions of the mutations found in the study.

Patient Number	Exon/Intron (NG_009018.1)	DNA Mutation gDNA Level (NG_009018.1)	DNA Mutation cDNA Level (NM_000267.3)	Predicted Protein (NP_000258.1)	Mutation Type	Variant Effect	References
NF.01	IVS11	g.29530107A > G	c.1260 + 1604A > G	p.N420_S421insLTT* (also noted as p.S421LfsX4)	Substitution	Splicing (deep intronic mutation)	Valero et al. [24]; Sabbagh et al. [27]; van Minkelen et al. [15]
NF.26 NF.27	E43	g.29664504C > G	c.6483C > G	p.Y2161*	Substitution	Splicing ^b^	Ars et al. [28]
NF.42	E37	g.29653014dup	c.4949dupA	p.Y1650*	Duplication	Frameshift truncation	Mattocks et al. [14]; van Minkelen et al. [15]
NF.54	E2	g.29483125del	c.185delT	p.L62*	Deletion	Frameshift truncation	Forzan [13]
NF.63	IVS3	g.29486116G > A	c.288 + 5G > A	p.(?)	Substitution	Splicing	ND
NF.78	E54	g.29684347del	c.7867delG	p.A2623Qfs*35	Deletion	Frameshift truncation	ND
NF.83	E21	g.29556173T > G	c.2540T > G	p.L847R	Substitution	Missense	van Minkelen et al. [15]; De Luca et al. [5]; Cali et al. [21]
NF.85 NF.84 NF.14	E10	g.29528131T > C	c.1139T > C	p.L380P	Substitution	Missense	van Minkelen et al. [15]
NF.91 NF.87	IVS21	g.29556484G > A	c.2850 + 1G > A	p.(?)	Substitution	Splicing	Ars et al. [28] van Minkelen et al. [15]
NF.96	IVS45	g.29665720A > T	c.6757 − 2A > T	p.(?)	Substitution	Splicing	Pasmant et al. [3]

p.(?): Effect on protein level not known with certainty; ND: mutation not described until now; the mutations described by van Minkelen et al. [15] have been deposited in Leiden Open Variation Database (LOVD); ^a^ Nonsense mutation, as predicted by Alamut^®^ Visual. It has a splicing alteration effect, causing an exon skipping, demonstrated at mRNA level by Ars et al. [28].

## Supplementary Materials

The following are available online at www.mdpi.com/2073-4425/7/12/133/s1, Table S1: Alignment parameters of the reads, Table S2: Mean coverage of *NF1* exons and introns among 12 patients.
