# Supplementary material for: Hybridization Capture-Based Next-Generation Sequencing to Evaluate Coding Sequence and Deep Intronic Mutations in the NF1 Gene
Source: Genes (Basel). 2016 Dec 17;7(12):133. doi: 10.3390/genes7120133 (PMC5192509; doi:10.3390/genes7120133)
Supplement: Supplementary file 1 [file genes-07-00133-s001.pdf]

Supplementary Materials: Hybridization Capture-Based Next-Generation Sequencing to Evaluate Coding Sequence and Deep Intronic Mutations in the *NF1* Gene

Karin Soares Cunha, Nathalia Silva Oliveira, Anna Karoline Fausto, Carolina Cruz de Souza, Audrey Gros, Thomas Bandres, Yamina Idrissi, Jean-Philippe Merlio, Rodrigo Soares de Moura Neto, Rosane Silva, Mauro Geller and David Cappellen

Table S1. Alignment parameters of the reads.

Patient Number	Run (Chip)	Bases	Q ≥ 20 Bases	Number of Reads	Mean Read Length (bp)	On Target More or Equal than 1×	On Target More or Equal than 30×	Average Depth of Coverage	Exon Coverage NM_000267	Total Variants
NF.78	1 (314)	145,076,360	118,026,221	1 263 774	114	99.6%	98.1%	470	99%/374×	182
NF.85	1 (314)	232,374,262	186,724,457	1,755,403	132	99.7%	92.2%	55	98%/51×	262
**Average (run 1)**		**188,725,311**	**152,375,339**	**1,509,196**	**123**	**99.7%**	**95.2%**	**263**	**99%/212×**	**222**
NF.54	2 (316)	39,799,495	32,415,569	346,595	115	99.6%	92.1%	74	97%/66×	254
NF.63	2 (316)	42,443,972	34,226,190	352,731	120	99.6%	94.3%	93	98%/80×	301
NF.84	2 (316)	50,298,157	40,355,523	390,420	129	99.8%	95.4%	113	99%/104×	275
NF.91	2 (316)	37,769,883	30,824,732	311,817	121	99.5%	87.6%	52	98%/48×	185
NF.96	2 (316)	67,269,612	52,670,930	495 396	136	99.9%	97.9%	150	99%/142×	157
**Average (run 2)**		**47,516,224**	**38,098,589**	**376,635**	**124**	**99.7%**	**93.5%**	**96**	**98.2%/88×**	**234**
NF.1	2 (318)	171,879,438	148,173,440	907,487	189	99.8%	99.4%	241	98%/242×	241
NF.19	2 (318)	150,777,557	127,627,392	675,954	203	99.8%	99.3%	448	98%/464×	271
NF.26	2 (318)	139,887,579	120,030,143	757,283	184	99.9%	98.3%	83	98%/81×	288
NF.83	2 (318)	128,053,076	110,230,360	636,802	201	99.9%	98.7%	103	98%/102×	288
NF.42	2 (318)	188,942,506	160,961,446	940,809	200	99.8%	99.4%	245	98%/250×	147
**Average (run 3)**		**155,908,031**	**133,404,556**	**796,662**	**195**	**99.8%**	**99.0%**	**224**	**98%/228×**	**247**
**Total Average (run 1 2 and 3)**		**116,214,325**	**96,855,534**	**773,477**	**154**	**99.7%**	**96.0%**	**177**	**98%/167×**	**238**

Table S2. Mean coverage of neurofibromatosis (*NF1*) exons and introns among 12 patients.

Location	Coverage (×)	Location	Coverage (×)	Location	Coverage (×)
Exon 1	9.5	IVS 20	161.4	Exon 40	155.3
IVS 1	146.9	Exon 21	210.7	IVS 40	90.6
Exon 2	205.3	IVS 21	228.4	Exon 41	201.6
IVS 2	167.7	Exon 22	147.9	IVS 41	152.7
Exon 3	160.0	IVS 22	150.8	Exon 42	194.0
IVS 3	154.3	Exon 23	100.7	IVS 42	160.4

Exon 4	196.7	IVS 23	220.0	Exon 43	154.2
IVS 4	175.8	Exon 24	185.7	IVS 43	80.6
Exon 5	180.9	IVS 24	138.4	Exon 44	77.0
IVS 5	74.7	Exon 25	116.5	IVS 44	46.1
Exon 6	138.8	IVS 25	146.8	Exon 45	138.9
IVS 6	159.5	Exon 26	159.1	IVS 45	94.1
Exon 7	130.5	IVS 26	130.3	Exon 46	108.7
IVS 7	179.0	Exon 27	204.7	IVS 46	152.3
Exon 8	139.1	IVS 27	177.9	Exon 47	154.8
IVS 8	125.2	Exon 28	205.7	IVS 47	166.3
Exon 9	168.5	IVS 28	175.3	Exon 48	218.8
IVS 9	210.3	Exon 29	191.9	IVS 48	110.4
Exon 10	180.4	IVS 29	140.2	Exon 49	120.5
IVS 10	188.4	Exon 30	246.0	IVS 49	175.9
Exon 11	126.4	IVS 30	168.8	Exon 50	165.7
IVS 11	159.9	Exon 31	194.4	IVS 50	140.6
Exon 12	247.2	IVS 31	141.2	Exon 51	104.6
IVS 12	144.1	Exon 32	189.0	IVS 51	155.9
Exon 13	68.5	IVS 32	85.4	Exon 52	152.5
IVS 13	167.2	Exon 33	124.7	IVS 52	145.3
Exon 14	199.7	IVS 33	158.9	Exon 53	223.7
IVS 14	126.5	Exon 34	215.8	IVS 53	162.1
Exon 15	114.8	IVS 34	155.7	Exon 54	227.4
IVS 15	153.0	Exon 35	180.2	IVS 54	193.0
Exon 16	139.9	IVS 35	94.0	Exon 55	171.2
IVS 16	160.8	Exon 36	176.4	IVS 55	187.0
Exon 17	166.2	IVS 36	160.6	Exon 56	280.4
IVS 17	157.5	Exon 37	190.1	IVS 56	192.9
Exon 18	215	IVS 37	159.9	Exon 57	223.4
IVS 18	210.5	Exon 38	244.7	IVS 57	152.1
Exon 19	166.1	IVS 38	184.8	Exon 58	161.5
IVS 19	119.6	Exon 39	180.3		
Exon 20	147.5	IVS 39	128.1		
